# Transsynaptic viral tools in neural circuit analysis: from anatomical mapping to functional interrogation

**DOI:** 10.3389/fncir.2026.1893057

**Published:** 2026-07-22

**Authors:** Zsolt Boldogkői, Gábor Torma, Zsolt Csabai, Zoltán Máté, Rebeka Fekete, Máté Mizik, Ferenc Erdélyi, Dóra Tombácz, Ádám Dénes

**Affiliations:** 1 Department of Medical Biology, Albert Szent-Györgyi Medical School, University of Szeged, Szeged, Hungary; 2 Medical Gene Technology Unit, HUN-REN Institute of Experimental Medicine, Budapest, Hungary; 3 Laboratory of Neuroimmunology, HUN-REN Institute of Experimental Medicine, Budapest, Hungary

**Keywords:** adeno-associated virus, fluorescent activity marker, inducible systems, neural tracing, optogenetics, pseudorabies virus, rabies virus

## Abstract

The application of transsynaptic viruses has transformed neural circuit analysis, enabling increasingly precise mapping of neuronal connectivity. These approaches can be broadly divided into polysynaptic and monosynaptic strategies. Polysynaptic retrograde tracers based on pseudorabies virus, including the classically selected Bartha strain and genetically engineered retrograde variants have provided important insights into multisynaptic circuit organization in rodents. For polysynaptic anterograde tracing, herpes simplex virus strain H129 and its derivatives have been used to map output pathways, with later genetic modifications improving detectability and experimental control. Over the past few years, growing attention has shifted toward monosynaptic tracing based on engineered rabies virus platforms used in combination with adeno-associated virus helper systems, which enable genetically restricted spread to direct presynaptic partners. Viral tracing is increasingly integrated with functional approaches, including calcium and voltage imaging, optogenetics, chemogenetics, and recombinase-based genetic strategies, thereby extending circuit analysis from anatomical mapping to causal interrogation of the function of defined neuronal populations. Looking forward, improved control of polysynaptic spread, together with large transgene capacity, suitability for tracing hierarchical circuit architecture, and the ability to incorporate functional readouts, is expected to revive interest in multisynaptic tracing.

## 1 Introduction

**Figure 1:**
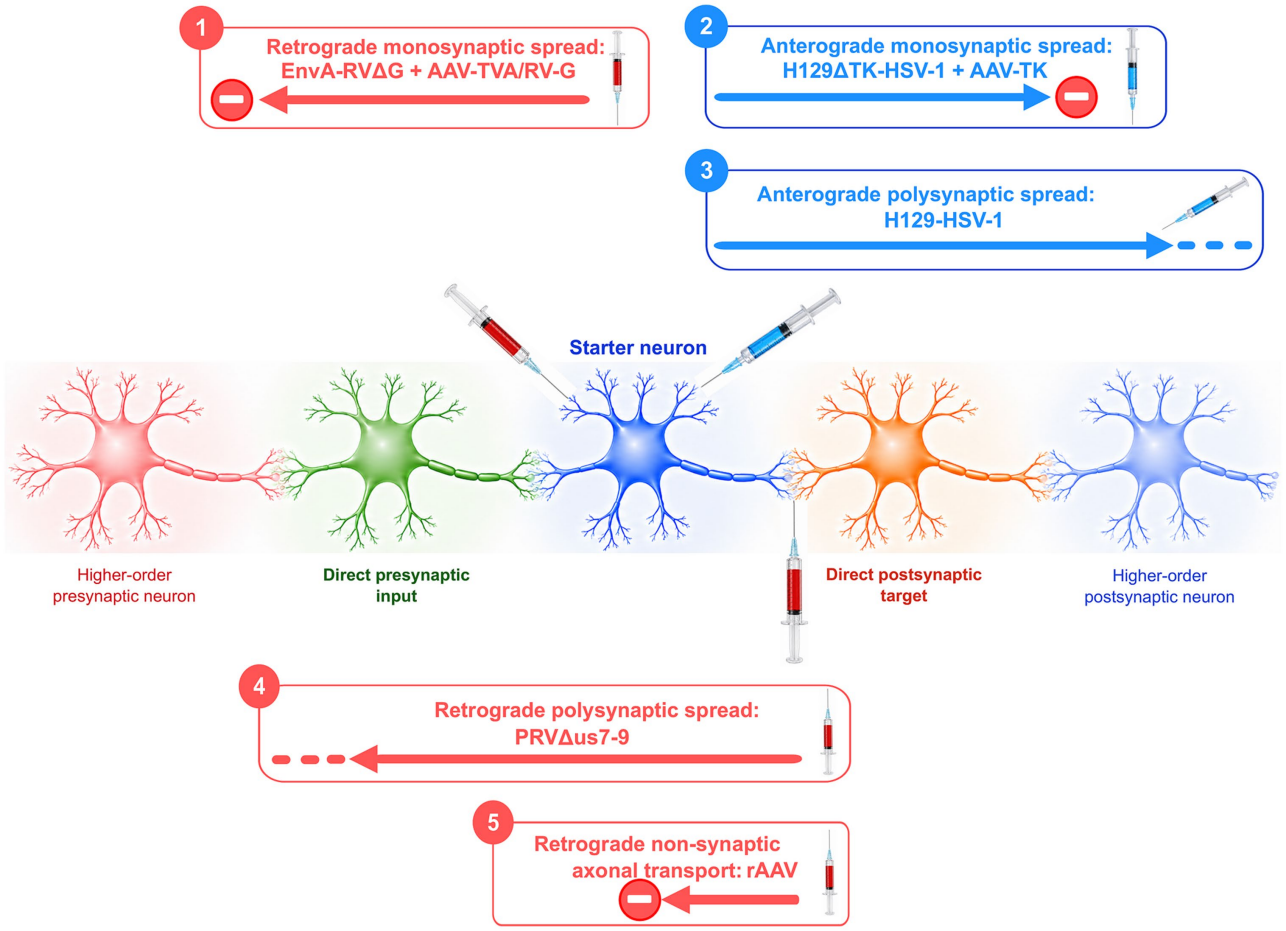
Schematic overview of viral transsynaptic tracing strategies. The figure illustrates the principal virus-based approaches for mapping neural circuit connectivity, organized around a central starter neuron (blue) and its synaptic partners. Five strategies are depicted, differentiated by their direction of spread (retrograde vs. anterograde) and the number of synaptic steps traversed (monosynaptic vs. polysynaptic). (1) Retrograde monosynaptic spread (EnvA-RVΔG + AAV-TVA/RV-G): EnvA-pseudotyped glycoprotein-deleted rabies virus, complemented by AAV-delivered TVA and RV-G in the starter population, spreads retrogradely across a single synaptic step to label only direct presynaptic inputs (green neuron). The minus sign indicates that further transsynaptic spread is blocked in the absence of RV-G in presynaptic partners. (2) Anterograde monosynaptic spread (H129ΔTK-HSV-1 + AAV-TK): Thymidine kinase (TK) gene-deleted HSV-1 strain H129, complemented by AAV-delivered TK in the starter population, spreads anterogradely to label only direct postsynaptic targets (orange neuron). The minus sign indicates restriction to a single anterograde synaptic step. (3) Anterograde polysynaptic spread (H129-HSV-1): The H129 strain of HSV-1 spreads anterogradely across multiple synaptic stations, labeling direct postsynaptic targets and higher-order postsynaptic neurons (dashed arrow). (4) Retrograde polysynaptic spread (PRVΔus7-9): Attenuated pseudorabies virus spreads retrogradely across multiple synaptic stations, labeling both direct presynaptic inputs and higher-order presynaptic neurons (red neuron, dashed arrow). Retrograde directionality is conferred by deletions in the *us7*, *us8*, and *us9* genes, which impair anterograde axonal sorting and transmission; these deletions are present in the classical Bartha strain and have been introduced independently into other PRV variants by targeted gene engineering. (5) Retrograde non-transsynaptic axonal transport (rAAV): Retrograde-optimized AAV is taken up by axon terminals and transported retrogradely to the cell body without crossing synapses, enabling genetic access to projection-defined neurons without transsynaptic labeling. Syringes indicate the site and composition of viral injections into the starter neuron region. Neuronal colors denote distinct connectivity-defined populations: blue, starter neuron; green, direct presynaptic input; red, higher-order presynaptic neuron; orange, direct postsynaptic target; light blue, higher-order postsynaptic neuron.

Deciphering the synaptic architecture of neural circuits—the structural foundation of brain organization—has driven the development of neurotropic virus-based transneuronal tract-tracing tools. Historically, these approaches began with polysynaptic methods to reveal higher-order connectivity (Enquist and Card, 2003; Boldogköi et al., 2004), before monosynaptic and genetically restricted systems became available. These systems share a common operational logic: the virus enters neurons, expresses its genes along with any transgenic cargo, and spreads to synaptically connected neurons, thereby labeling the circuit. While polysynaptic tracers propagate serially across multiple connections to map higher-order circuit organization, monosynaptic systems restrict spread to a single synaptic step—labeling only direct presynaptic inputs in the retrograde direction, or direct postsynaptic targets in the anterograde direction (Figure 1). Classical polysynaptic systems based on alphaherpesviruses play an important role in revealing multisynaptic circuit organization: pseudorabies virus (PRV) strains selected or engineered for retrograde spread provide reliable labeling across multiple synaptic stations, while herpes simplex virus type 1 (HSV-1) strain H129 and its derivatives enable anterograde output tracing (Sun et al., 1996; Zeng et al., 2017). Monosynaptic retrograde tracing is most commonly based on engineered rabies virus (RV) systems in which deletion of the viral glycoprotein (RV-G) gene produces the spread-deficient vector RVΔG, which cannot undergo onward transsynaptic spread unless RV-G is provided *in trans* by a complementing adeno-associated virus (AAV) vector (AAV-RV-G). The helper vectors are injected directly into the starter region and transduce local neurons rather than crossing synapses, thereby providing tumor virus receptor A (TVA) and RV-G to the target starter population. Pseudotyping of RVΔG with EnvA (avian sarcoma and leukosis virus envelope protein A) restricts initial infection to TVA-expressing cells: both EnvA and TVA are of avian origin and are absent from mammalian neurons, so the virus can only enter cells that have been engineered to express TVA. After replication in these starter cells, complemented rabies virions are released at synaptic contacts and taken up by presynaptic axon terminals contacting the starter-cell soma or dendrites, from which they are transported retrogradely to the input neurons (Wickersham et al., 2007b). Anterograde monosynaptic strategies include engineered HSV-1 amplicon systems (Xu et al., 2020; Fischer et al., 2023), AAV-mediated transsynaptic tagging (Zingg et al., 2017, 2020), and synthetic protein-based platforms that transfer recombinase payloads across synapses without viral replication (Rivera et al., 2025). In recent years, the field has shifted toward monosynaptic and directionally restricted systems with improved safety and experimental control, although polysynaptic tracing retains distinct value for mapping higher-order circuit architecture—a role that may expand further as innovations in conditional spread, cell-type-specific complementation, and exploitation of the large transgene capacity of herpesvirus genomes continue to advance. Viral circuit analysis is increasingly integrated with complementary technologies, including tissue clearing (Chung et al., 2013), single-cell transcriptomics (Patiño et al., 2024; Zhang et al., 2024), molecular barcoding approaches (Chen et al., 2019), calcium and voltage imaging (Boldogkoi et al., 2009; Gong et al., 2015), optogenetics (Boyden et al., 2005), chemogenetics (Alexander et al., 2009), recombinase-based (Sauer and Henderson, 1988) and inducible (Feil et al., 1997) genetic systems. In this review, we first survey the major poly- and monosynaptic tools currently available for circuit analysis, discuss their applications, limitations, and evolving roles, and then examine how integration with functional approaches—including calcium and voltage imaging, optogenetics, and chemogenetics—alongside molecular and high-throughput strategies, is transforming viral tracing from a purely anatomical mapping strategy into a multimodal platform. The main operational features, experimental strengths, and limitations of the principal viral and virus-enabled platforms are summarized in Table 1.

**Table 1:** Comparison of major viral and virus-enabled platforms for neural circuit mapping.

Platform	Spread	Targeting / control	Best use	Key caveats
Pseudorabies virus: Bartha and genetically engineered retrograde strains	Retrograde Polysynaptic	Injection-defined Survival-time progression Recombinant reporters	Multisynaptic upstream mapping Peripheral organ–CNS/autonomic pathways Hierarchical circuit architecture	Cytopathic Short experimental window Synaptic order inferred indirectly Late nonspecific spread possible
Herpes simplex virus type 1: H129-based tracers	Anterograde-biased Polysynaptic	Injection-defined Strain-dependent bias	Downstream/output pathway mapping Multisynaptic output architecture	Highly cytopathic Delayed retrograde spread can occur Synaptic order difficult to resolve
Engineered H129 monosynaptic systems	Anterograde Monosynaptic/restricted	TK- or UL6-deletion Cre-dependent AAV complementation	Direct output mapping Genetically defined starter cells	Technically complex Helper leakiness must be controlled Incomplete restriction possible
First-generation RVΔG systems	Retrograde Monosynaptic	EnvA–TVA targeting RV-G supplied in starter cells AAV helper vectors	Direct presynaptic input mapping Defined starter populations	Substantial toxicity Limited time window Helper leakiness and starter-cell number affect interpretation
Improved RV systems: CVS-N2c, SiR, RVΔGL, RVΔL	Retrograde Monosynaptic	Modified rabies backbones G/L/N complementation Self-inactivation	More efficient or longer-term input mapping Functional imaging of traced circuits	More complex production Strict genomic QC needed System-specific validation required
Barcoded RV platforms	Retrograde Monosynaptic (RV-based)	RV-based tracing Molecular barcode libraries	High-throughput input mapping Connectivity linked to single-cell identity	Requires sequencing readout Barcode diversity and contamination must be controlled
AAV1/AAV9 transsynaptic tagging	Mainly anterograde Monosynaptic-like	AAV-mediated transfer Cre/Flp-compatible logic	Low-toxicity output tagging Long-term genetic access Intersectional strategies	Lower efficiency/synaptic specificity than RV/HSV Possible retrograde or nonsynaptic confounds
Synthetic transsynaptic systems, e.g., ATLAS	Anterograde Monosynaptic	AAV-delivered protein transfer Activity-dependent labeling possible	Low-toxicity labeling of active postsynaptic partners	Newer platform Less established than classical viral systems
TRIO/cTRIO projection-defined rabies tracing	Retrograde Monosynaptic (RVΔG-based) Input–output circuit mapping	Retrograde Cre delivery to projection-defined neurons Cre-dependent AAV-TVA/RV-G helpers cTRIO adds Flp/cell-type intersection	Mapping direct presynaptic inputs to neurons defined by projection target Input–output organization Cell-type- and projection-restricted starter populations	Multi-virus/multi-site design Requires precise injections and efficient retrograde delivery Helper leakiness/starter-cell number affect interpretation Mainly maps inputs to projection-defined cells

The table summarizes the main platforms discussed in this review according to their spread profile, targeting strategy, optimal use, and key limitations. Rather than identifying a universally optimal tracer, the table highlights the experimental trade-offs that determine whether a platform is better suited for direct input mapping, output mapping, multisynaptic circuit architecture, long-term functional access, or molecularly resolved circuit analysis.

## 2 Classical polysynaptic tracers

### 2.1 Principles of polysynaptic tracing

Alphaherpesviruses used for polysynaptic tracing infect neurons, replicate in the nucleus, and propagate through connected neuronal networks. Wild-type strains are capable of both anterograde and retrograde spread, whereas tracer strains are selected or engineered to favor one direction (Pomeranz et al., 2005). For circuit analysis, viral spread must be sufficiently slow to allow temporal inference of synaptic order, yet sufficiently efficient to label higher-order neurons within an experimentally tolerable survival window. This balance is typically achieved by attenuation, which delays cytopathic effects and prolongs the interval during which transsynaptic progression can be resolved. Ideally, viral spread should remain largely confined to synaptically connected neurons, rather than reflecting nonspecific extracellular dissemination of virions, which would compromise the interpretability of the tracing signal. Attenuated strains may partly support this requirement by delaying cytopathic damage, thereby extending the interval during which local microglial containment may help limit parenchymal viral spillover (Fekete et al., 2018). A further essential requirement is the expression of a robust and readily detectable marker. Early studies relied on immunohistochemical detection of viral antigens in fixed tissue. Recombinant strains carrying fluorescent reporter genes enabled direct visualization of infected neurons and, importantly, made it possible to identify circuit-defined neurons for electrophysiological and optical functional analysis in living brain slices and *ex vivo* retinal preparations (Boldogköi et al., 1999, 2002; Smith et al., 2000).

### 2.2 Alphaherpesviruses

The Bartha strain of PRV, originally developed as a live attenuated vaccine (Bartha, 1961), became a foundational transsynaptic tracer. Deletions in the us7/gI, us8/gE, and us9 genes—present in Bartha and related engineered PRV strains—impair anterograde axonal sorting and transmission, biasing spread toward the retrograde direction (Lyman et al., 2007; Curanović et al., 2009; Kratchmarov et al., 2013). Mechanistically, the Us7/gI–Us8/gE–Us9 complex is required for efficient recruitment of kinesin motors, including the kinesin-3 motor KIF1A, to newly assembled viral particles at the trans-Golgi network, thereby promoting axonal sorting and anterograde transport toward presynaptic terminals (Kramer et al., 2012; Scherer et al., 2020). Loss of these viral proteins therefore reduces anterograde delivery of progeny virions and shifts the apparent directionality of spread toward the retrograde route. Importantly, this directionality should be interpreted as a strong bias rather than an absolute property. Residual labeling in the non-preferred direction can occur depending on viral dose, neuronal subtype, injection site, and survival time, and late cytopathic damage may further increase nonspecific extracellular release of virions (Card et al., 1990; Fekete et al., 2018). Because PRV spreads progressively across synapses, the time allowed for viral propagation determines how many synaptic steps are labeled: short intervals restrict labeling to direct presynaptic partners, whereas longer intervals allow the virus to reach successively higher-order neurons (Strack et al., 1989; Card et al., 1990; Enquist and Card, 2003). This temporal logic was further refined by genetically timed PRV variants carrying two reporter genes that generated an early green and a delayed red signal through differences in both expression timing and fluorophore maturation, allowing color composition to report relative infection time and thereby helping resolve synaptic order between connected regions without relying solely on timing of viral infection (Boldogkoi et al., 2009). The fluorescent reporter-based toolbox was further extended by multicolor Rainbow PRVs, in which spectrally distinct recombinant viruses injected into different targets enabled fine spatial organization in upstream centers to be resolved and overlapping retrograde pathways to be disentangled (Boldogkoi et al., 2009). Using this approach, the fine-scale internal organization of a nucleus—such as in the locus coeruleus—can be resolved by revealing how neurons receiving input from distinct targets are spatially arranged within it (Boldogkoi et al., 2009).

HSV-1 was originally introduced as a retrograde transneuronal tracer by Ugolini and colleagues, who demonstrated its synaptic transfer from peripheral motor and sensory nerves to higher-order central neurons (Ugolini et al., 1987; McLean et al., 1989). The importance of HSV-1 for circuit mapping was expanded by the recognition that strain H129 shows a strong anterograde bias, enabling multisynaptic tracing of output pathways from a defined injection site (Sun et al., 1996). The anterograde bias of H129 is thought to arise from preferential axonal sorting and kinesin-1-dependent transport of viral capsids toward axon terminals, which promotes transmission to downstream postsynaptic neurons, although the precise molecular basis remains incompletely understood (Zeng et al., 2017; Dong et al., 2020). However, this preference is relative rather than absolute: H129 can also undergo temporally delayed retrograde spread, especially when survival times are extended or high viral titers are used (Wojaczynski et al., 2015). Thus, H129-based labeling should be interpreted in relation to the experimental time window, because early labeling is more likely to reflect the intended anterograde progression, whereas late labeling may include retrograde or nonspecific components. Together with the cytopathic and polysynaptic nature of classical H129 tracing, these limitations motivated the development of engineered H129-derived systems with improved control over synaptic order, toxicity, and starter-cell specificity.

### 2.3 Applications of viral polysynaptic tracers

Alphaherpesvirus-based retrograde tracing has been applied across a wide range of neural systems. In autonomic neuroscience, PRV was used to map the central control of visceral organs—including the stomach, adrenal gland, kidney, bone marrow, spleen, thymus, ovary, and mammary gland—as well as the postnatal assembly of limbic-autonomic connections (Strack et al., 1989; Card et al., 1990; Gerendai et al., 1998; Rinaman et al., 1999, 2000; Dénes et al., 2005). In sensory and neuroendocrine systems, PRV has illuminated hippocampal circuits, retinal networks of melanopsin-containing ganglion cells, the multisynaptic organization of the suprachiasmatic nucleus and its connections to arousal and reward circuits, and pinealocyte–retinal pathways (Aston-Jones et al., 2001; Pickard et al., 2002; Sík et al., 2006; Viney et al., 2007; Csáki et al., 2021). Applications to motor and respiratory systems have identified serotonergic premotor neurons mediating thermoregulation, brainstem circuits coordinating sympatho-motor responses, respiratory motoneuron control, and motor cortical reorganization after facial denervation (Billig et al., 2000; Horváth et al., 2002; Kerman et al., 2003; Nakamura, 2004; Tóth et al., 2006). PRV infection has also served as a model for studying glial responses to neurotropic viruses, revealing that microglial P2Y12-dependent recruitment and phagocytosis can limit viral spread within the CNS (Fekete et al., 2018).

Collectively, these studies illustrate the particular value of polysynaptic herpesvirus tracers: rather than simply identifying direct inputs or outputs, they reveal the hierarchical organization of multi-step pathways linking peripheral targets, autonomic and neuroendocrine centers, sensory systems, and motor or respiratory control networks.

## 3 Monosynaptic retrograde tracing with rabies virus

### 3.1 Principles and first-generation systems

The major conceptual breakthrough for monosynaptic retrograde tracing came with the deletion of the G gene from the RV genome—a modification that renders the virus spread-deficient (Wickersham et al., 2007b). The resulting vector, RVΔG, can enter neurons and express transgenic cargo, but cannot produce new infectious particles on its own. To further restrict initial infection to a defined starter population, RVΔG is pseudotyped with EnvA, which binds exclusively to TVA delivered to the target population by AAV helper vectors. In this system, starter neurons are first transduced with AAV helper vectors encoding TVA and RV-G, either on separate vectors (AAV-TVA and AAV-RV-G) or on a single bicistronic vector (AAV-TVA/RV-G; e.g., FLEX-TVA-2A-oG). After sufficient helper expression, EnvA-RVΔG is introduced; following retrograde spread to presynaptic partners, which lack helper-derived RV-G, the virus cannot spread further.

Although this design operationally restricts labeling to first-order presynaptic neurons, “transsynaptic” spread should not be interpreted as proof of strictly synapse-exclusive transfer. The precise membrane events that allow RVΔG to enter presynaptic elements remain incompletely resolved, and labeling efficiency can be influenced by synapse number, local membrane proximity, viral titer, and cell-type-dependent factors (Beier et al., 2015; Rogers and Beier, 2021; Patiño et al., 2022a).

In most applications, TVA and RV-G are placed under Cre recombinase (Cre)-dependent FLEX (flip-excision cassette)/DIO (double-floxed inverted orientation) control, so that only neurons expressing Cre become starter competent. Cre is most commonly provided by knock-in driver lines, in which the Cre gene is inserted under the control of an endogenous cell-type-specific promoter (e.g., Pvalb-Cre, Sst-Cre, or Vip-Cre lines), but bacterial artificial chromosome (BAC) transgenic lines or AAV vectors carrying short cell-type-specific promoters can also be used (Wall et al., 2010). The most commonly used EnvA-RVΔG strains are SAD-B19ΔG, which offers straightforward production and high transgene expression levels, and CVS-N2cΔG, which demonstrates approximately 10–20-fold higher transsynaptic transfer efficiency and improved neuronal viability (Wickersham et al., 2010; Reardon et al., 2016). First-generation rabies tracers, however, remain substantially toxic: starter neurons, supporting full viral replication, die rapidly, while presynaptic input neurons typically survive only one to two weeks before viral gene expression causes cell death, thereby limiting their use in long-term experiments (Wickersham et al., 2007a; Ciabatti et al., 2017).

### 3.2 Second- and third-generation rabies virus vectors

To address toxicity, several new generations of monosynaptic RV vectors have been developed (Chatterjee et al., 2018; Jin et al., 2024). One strategy, exemplified by the self-inactivating RV (SiR), reduces toxicity by modifying the viral nucleoprotein (N) with a Pro-Glu-Ser-Thr-rich sequence (PEST) domain, a short amino acid sequence that targets N for rapid proteasomal degradation (Ciabatti et al., 2017). Because N is essential for viral transcription and replication, this modification limits the duration of viral activity rather than allowing sustained replication and the associated cytotoxicity. Importantly, monosynaptic restriction in SiR is still conferred by the same ΔG-based logic used in first-generation rabies systems: helper-dependent complementation of RV-G is restricted to starter cells, so although a brief initial window of viral activity is sufficient for recombinase expression and spread to first-order presynaptic partners, these secondary infected neurons do not express helper-derived RV-G and therefore cannot support further transsynaptic spread (Wickersham et al., 2007b; Callaway and Luo, 2015; Ciabatti et al., 2017). During this early window, the SiR genome expresses a site-specific recombinase—either Cre or its functional analogue flippase recombinase (Flp), which recognizes FRT (flippase Recognition Target) rather than loxP (locus of crossing-over in bacteriophage P1) sites—allowing excision of a transcriptional STOP cassette from a co-delivered AAV reporter construct and thereby stable activation of reporter or effector transgenes. Because this recombination-mediated transgene expression persists after viral silencing, traced neurons remain accessible for long-term anatomical and functional analysis. The choice between Cre and Flp depends on which recombinase system is not already being used for starter cell targeting. Subsequent work, however, raised important concerns about the practical implementation of this strategy. Jin and colleagues found that a substantial fraction of viral particles in some SiR preparations had already lost the PEST modification during *in vitro* amplification, before injection (Jin et al., 2023). This occurs because variants lacking the PEST domain gain a selective advantage during production in packaging cells. These findings are not necessarily inconsistent with the *in vivo* stability reported by others (Ciabatti et al., 2023), because the two studies addressed different aspects of the system (Jin et al., 2023).

A complementary approach removes not only the G gene but also the viral polymerase gene L (RVΔGL double-deletion mutants). Because the viral polymerase is required for all transcription from the RV genome, its deletion abolishes cytotoxicity while reducing transgene expression to low but functionally useful levels—sufficient to trigger Cre- or Flp-mediated recombination in a reporter construct, since even trace amounts of recombinase can catalyze irreversible loxP or FRT site excision (Sauer and Henderson, 1988; Dymecki, 1996). These vectors were initially characterized as non-toxic retrograde tracers; subsequent work demonstrated that RVΔGL vectors can support monosynaptic tracing when both RV-G and RV-L are supplied *in trans* in starter cells, with over 90% of labeled neurons surviving 12-week longitudinal imaging periods with unperturbed visual response properties (Chatterjee et al., 2018; Jin et al., 2024). More recently, third-generation ΔL vectors—in which deletion of the polymerase gene alone is sufficient to abolish toxicity—achieve similar non-toxicity to RVΔGL vectors while growing to substantially higher titers, enabling far greater numbers of retrogradely labeled neurons *in vivo* and making routine large-scale tracing more feasible (Jin et al., 2023; Zhang et al., 2024).

### 3.3 Applications of rabies virus-based systems

Rabies virus-based monosynaptic tracing has enabled systematic mapping of direct presynaptic inputs to genetically, anatomically, or projection-defined neuronal populations across the brain. These approaches have revealed not only which neurons provide input to a given starter population, but also that different cell types and projection-defined neuronal populations can receive distinct patterns of long-range and local input (Callaway and Luo, 2015). For example, brain-wide RV tracing of cortical interneuron classes showed that parvalbumin-, somatostatin-, and VIP-expressing interneurons receive inputs from overlapping but quantitatively distinct sets of cortical, thalamic, and basal forebrain regions, demonstrating that inhibitory cell classes differ not only in their local circuit roles but also in their long-range input architecture (Wall et al., 2016). Projection-defined rabies strategies further revealed the input–output organization of dopaminergic neurons in the ventral tegmental area (VTA). In these experiments, these neurons are first defined by their downstream projection target, and rabies virus-based monosynaptic tracing is then used to identify the direct presynaptic neurons that provide input to each projection-defined population. This approach showed that VTA neurons projecting to different brain regions receive inputs from different upstream sources, linking their output target to their input organization (Beier et al., 2015). RV tracing has also been combined with whole-brain imaging to define brain-wide input organization for specific neuronal populations, including descending inputs onto spinal V1 interneurons and their molecularly distinct subsets (Chapman et al., 2025). Similar approaches have mapped inputs to basal forebrain calretinin-expressing neurons and linked these circuits to odor learning (Constantinescu et al., 2025).

## 4 Anterograde monosynaptic tools

### 4.1 Engineered H129-derived HSV-1 systems

Early H129-derived tracers were effective for anterograde tracing but exhibited polysynaptic spread, limiting their ability to identify direct output targets (Sun et al., 1996; Wojaczynski et al., 2015; Zeng et al., 2017). A first step toward monosynaptic restriction was achieved by deleting the thymidine kinase (TK) gene from the H129 genome and supplying TK *in trans* via a Cre-dependent AAV helper virus (AAV-TK), so that productive replication—and therefore transsynaptic spread—occurs only in the genetically defined starter cell population (Zeng et al., 2017). An earlier proof of principle for Cre-dependent anterograde transsynaptic tracing from genetically marked neurons was also established using a modified viral vector system (Lo and Anderson, 2011). More recently, H129 amplicon (H129Amp) systems have been developed to uncouple tracer expression from the full viral replication cycle. In these systems, the fluorescent reporter or functional transgene is carried by an HSV-1 amplicon, a minimal HSV-derived DNA vector that contains the cis-acting sequences required for replication and packaging but lacks viral protein-coding genes. Because the amplicon cannot produce viral proteins on its own, replication and packaging functions are supplied in trans by a co-injected H129-derived helper virus in starter cells. In one implementation, this helper virus is TK-deficient and carries a floxed packaging signal (pacFlox). The amplicon expresses Cre, which excises the packaging signal from the helper genome, preventing further helper-virus encapsidation and favoring the production and release of reporter-carrying amplicon particles rather than helper virions (Xu et al., 2020; Xiong et al., 2022). As a result, amplicon particles can be transmitted from starter neurons to their direct postsynaptic targets, whereas continued helper-virus propagation is blocked, thereby limiting labeling largely to a single anterograde synaptic step. This design preserves strong labeling while improving control over spread, reducing toxicity, and shortening experiment duration from weeks to days (Xu et al., 2020; Xiong et al., 2022). H129Amp-based systems have therefore been particularly useful for identifying downstream postsynaptic targets from defined starter regions or from genetically selected starter-cell populations, such as neurons targeted by Cre-dependent helper systems. By enabling rapid anterograde monosynaptic labeling and supporting combinations with functional circuit analysis, these approaches move output mapping closer to experimentally manipulable postsynaptic populations (Xiong et al., 2022). An alternative strategy uses deletion of the viral genome packaging gene *ul6*, which encodes a protein required for viral DNA packaging, with Cre-dependent complementation by AAV to restrict spread to one anterograde synaptic connection (Fischer et al., 2023).

### 4.2 AAV-based transsynaptic approaches

AAVs lack any replication mechanism in neurons, yet certain serotypes—notably AAV1 and AAV9—can cross synapses following injection into a presynaptic region. Transfer appears to depend on synaptic vesicle release rather than active viral spread (Zingg et al., 2020), suggesting that AAV particles are co-released with neurotransmitter and taken up by postsynaptic neurons via endocytosis. Whether transfer is strictly confined to active synapses or can also occur by non-synaptic transcytosis—in which particles are passively transported across the axon membrane independently of synaptic activity—remains unresolved (Zingg et al., 2017). AAV-based transsynaptic tagging is generally less efficient and less strictly synapse-specific than RV or herpesvirus systems, and its potential for retrograde transport can complicate interpretation, especially in circuits with reciprocal connectivity (Zingg et al., 2017, 2020).

### 4.3 Synthetic transsynaptic systems

More recently, increasing effort has been directed toward transsynaptic methods in which the transfer between neurons is no longer mediated by viral replication itself (Talay et al., 2017; Rivera et al., 2025). As a proof of principle, ATLAS (Anterograde Transsynaptic Label based on Antibody-like Sensors) demonstrated that genetically defined presynaptic neurons can be used to label their postsynaptic partners through a non-replicating protein-based mechanism (Rivera et al., 2025). In this approach, presynaptic neurons produce a payload composed of two linked parts: an antibody-like binding domain, AMPA. FingR, which recognizes the extracellular region of the GluA1 subunit of AMPA receptors on postsynaptic neurons, and Cre recombinase. This payload is released from presynaptic terminals via synaptic vesicle exocytosis into the synaptic cleft, binds to GluA1 on the postsynaptic membrane, and is then taken up by endocytosis into the postsynaptic neuron. After internalization, Cre reaches the nucleus and activates a floxed reporter gene, thereby marking the postsynaptic cell (Rivera et al., 2025). Because the payload is released through synaptic vesicle fusion, ATLAS does not rely on viral replication and provides activity-dependent anterograde labeling, preferentially marking synapses active during the labeling window while reducing the toxicity associated with replicating viral tracers (Rivera et al., 2025). A related conceptual precedent is the trans-Tango system in Drosophila, which uses a similar ligand–receptor strategy for anterograde transsynaptic labeling (Talay et al., 2017). Beyond connectivity mapping itself, these tracing strategies can be integrated with complementary anatomical, molecular, functional, and causal readouts.

## 5 Strategies for conditional starter cell targeting

### 5.1 Recombinase-based systems

**Figure 2:**
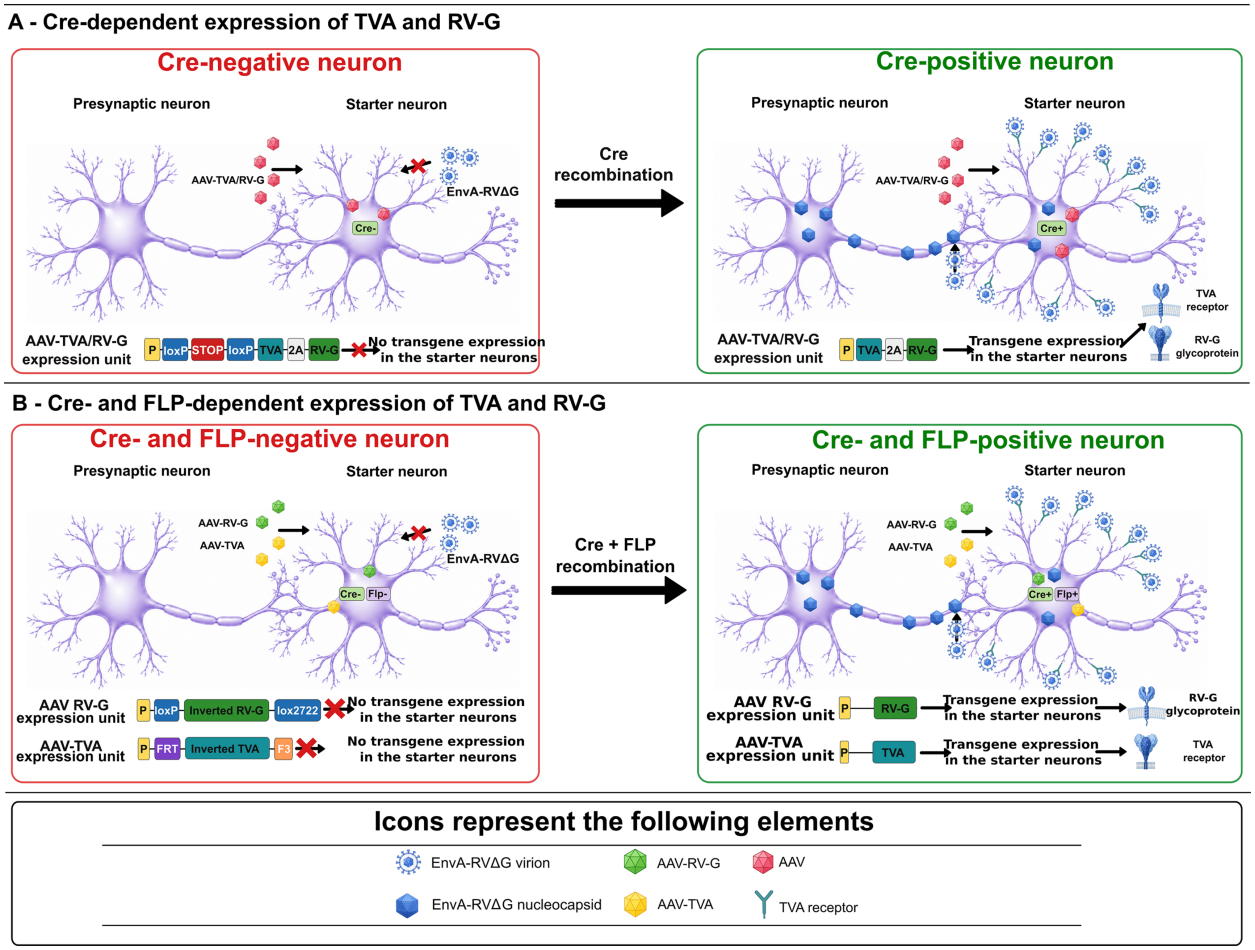
Recombinase-dependent systems. Both panels illustrate the conditions under which TVA and RV-G expression is activated, enabling starter cell competence for EnvA-RVΔG infection and monosynaptic retrograde tracing. Icons representing viral vectors and molecular components are defined in the figure key. **(A)** Cre-dependent expression of TVA and RV-G. A single bicistronic AAV helper vector (AAV-TVA/RV-G) carries TVA and RV-G linked by a 2A self-cleaving peptide within a FLEX cassette (P–loxP–STOP–loxP–TVA–2A–RV-G). In Cre-negative neurons (left), the STOP cassette prevents transcription and EnvA-RVΔG cannot infect the cell. In Cre-positive neurons (right), Cre-mediated excision of the STOP cassette allows TVA and RV-G expression, rendering the neuron competent for EnvA-RVΔG infection and monosynaptic tracing. **(B)** Cre- and Flp-dependent intersectional expression of TVA and RV-G. Two separate AAV helper vectors are used: AAV-RV-G carries RV-G in a Cre-dependent FLEX cassette (P–loxP–inverted RV-G–lox2722), and AAV-TVA carries TVA in a Flp-dependent fDIO cassette (P–FRT–inverted TVA–F3). In neurons lacking both recombinases (left), neither transgene is expressed and EnvA-RVΔG cannot infect. Only neurons co-expressing both Cre and Flp (right) activate both cassettes, producing RV-G glycoprotein and TVA receptor and becoming fully starter-competent. This intersectional AND-gate logic restricts starter cell identity to neurons satisfying two independent molecular criteria simultaneously.

A central challenge across viral circuit tracing approaches—RV-, HSV-1-, and AAV-based alike—is ensuring that labeling is confined to the intended starter population. The most widely used solution is to deliver TVA and RV-G via Cre-dependent AAV helper vectors, so that only neurons expressing Cre recombinase become competent starter cells (Sauer and Henderson, 1988; Wall et al., 2010); this strategy relies on the site-specific Cre/loxP recombination system originally established in mammalian cells (Sauer and Henderson, 1988). TVA and RV-G can be delivered on separate vectors or combined into a single bicistronic construct linked by a 2A self-cleaving peptide sequence—such as FLEX-TVA-2A-oG—which allows two independent proteins to be produced from a single mRNA through ribosomal skipping during translation. Because a large number of transgenic mouse lines express Cre in specific cell types, this approach provides broad and flexible access to virtually any genetically defined neuronal population (Figure 2A). Greater precision can be achieved through intersectional strategies, in which two recombinases—most commonly Cre and Flp—each control one component of the helper system via separate FLEX and fDIO (Flp-dependent DIO) cassettes, so that full starter cell competence requires both to be active simultaneously (Figure 2B). Only neurons co-expressing both Cre and Flp produce both helper components and become fully starter-competent; neurons expressing only one recombinase produce only one component and cannot initiate tracing. This design is not compatible with a single bicistronic 2A construct, because placing both components under the same recombinase would eliminate the AND-gate logic; packaging both cassettes into a single AAV is likewise precluded by the vector’s ~ 4.7 kb cargo capacity. The AND-gate specificity of the intersectional system depends entirely on the promoters driving Cre and Flp expression in the host cell, which are typically distinct: Cre is most commonly provided by a knock-in driver line under the control of a cell-type-specific endogenous promoter, while Flp is delivered either by a separate driver line crossed with the Cre line to yield double-transgenic animals, or by a retrograde AAV carrying a different cell-type- or region-specific promoter—the latter avoiding the need for additional breeding. Because the two recombinases are thus expressed under independent transcriptional control, only neurons satisfying both molecular criteria simultaneously become starter-competent—for example, belonging to a specific cell type and residing in a specific brain region (Dymecki, 1996; Fenno et al., 2014).

### 5.2 Pharmacologically controlled systems

**Figure 3:**
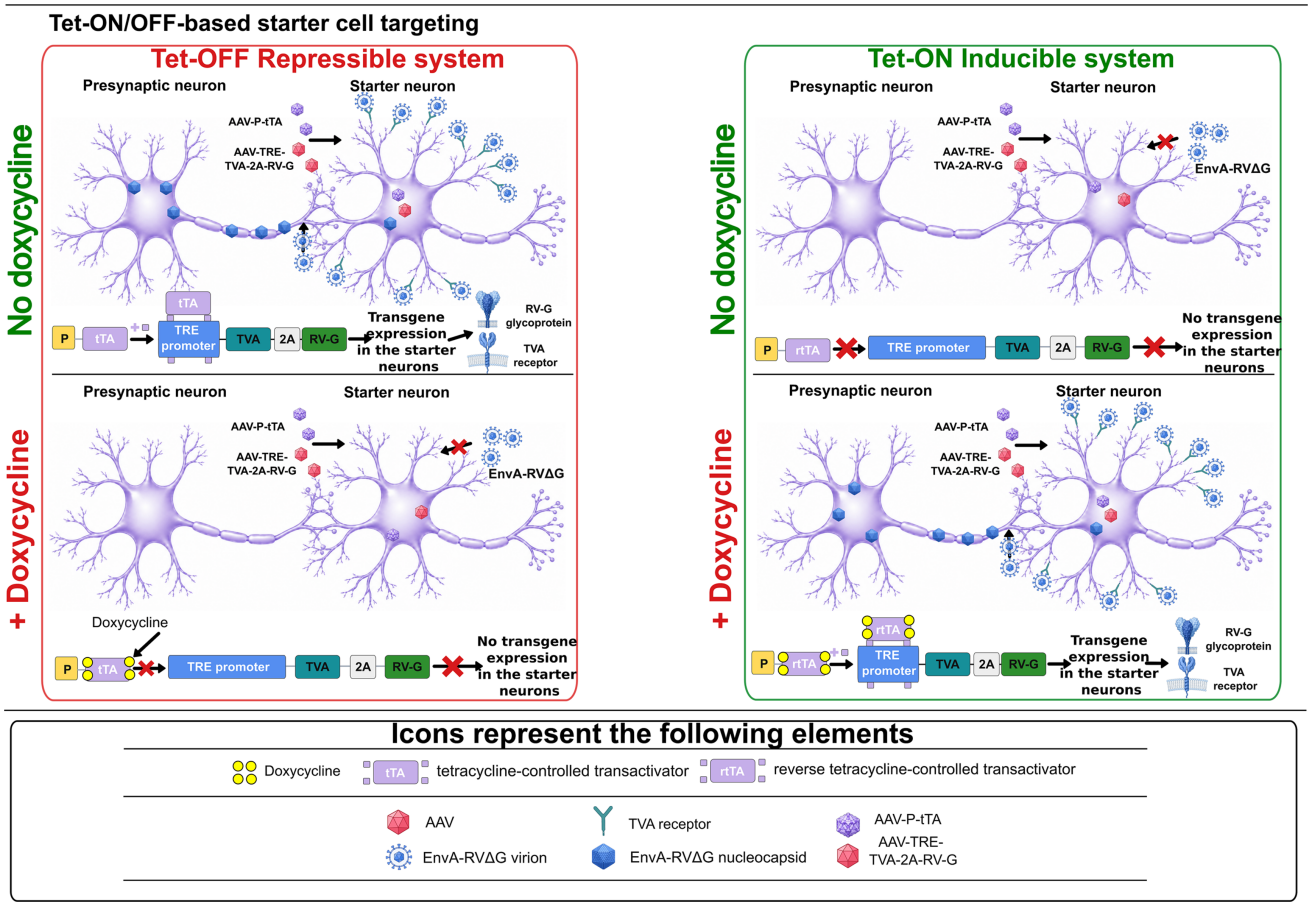
Pharmacologically controlled starter cell targeting. Tetracycline-regulated repressible and inducible systems. In the Tet-OFF system (left panels), AAV-P-tTA constitutively expresses the tetracycline-controlled transactivator (tTA), which binds the TRE (tetracycline response element) promoter in AAV-TRE-TVA-2A-RV-G and drives TVA and RV-G expression in the absence of doxycycline; doxycycline administration silences tTA binding and abolishes transgene expression. In the Tet-ON system (right panels), the reverse transactivator (rtTA) is inactive by default and binds the TRE promoter only upon doxycycline administration, switching on TVA and RV-G expression exclusively during the period of drug treatment. Together, these systems provide pharmacological control over the timing and duration of starter cell competence.

**Figure 4:**
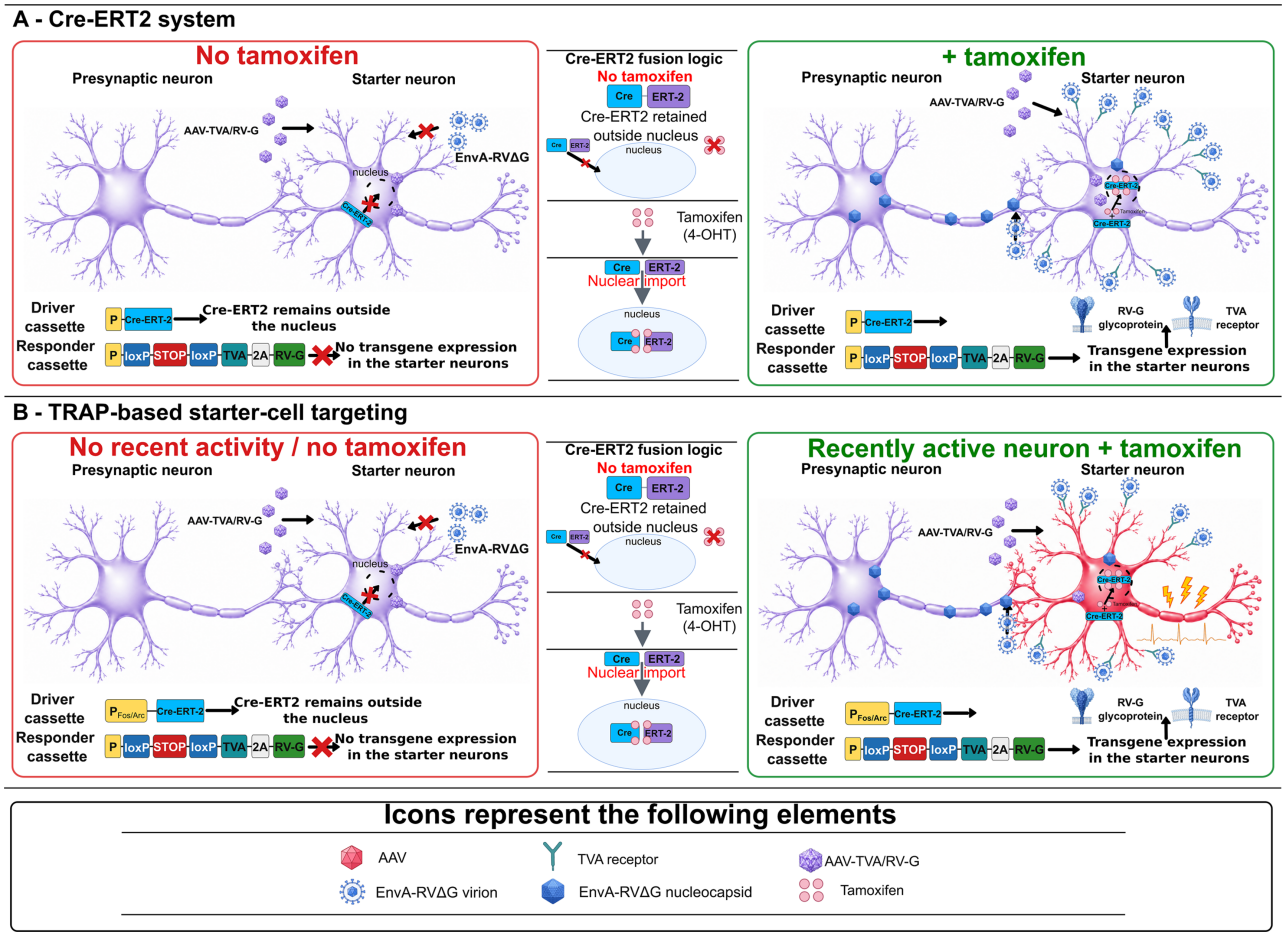
Tamoxifen- and TRAP-based starter-cell targeting. **(A)** Tamoxifen-inducible Cre-ERT2 system. The driver cassette, expressed by the neuron itself under a cell-type-specific or otherwise defined promoter, encodes Cre-ERT2, in which Cre recombinase is fused to a mutated estrogen receptor ligand-binding domain that no longer responds to endogenous estrogens but remains sensitive to tamoxifen or its active metabolite 4-hydroxytamoxifen (4-OHT). In the absence of tamoxifen, Cre-ERT2 is retained in the cytoplasm and the AAV-delivered responder/helper cassette remains inactive. The responder/helper cassette has the structure P–loxP–STOP–loxP–TVA–2A–RV-G. Upon tamoxifen administration and conversion to 4-OHT, Cre-ERT2 translocates to the nucleus, excises the STOP cassette, and activates TVA and RV-G expression, rendering the neuron competent for EnvA-RVΔG infection and monosynaptic tracing. **(B)** TRAP-based activity-dependent starter-cell targeting. In TRAP, an immediate early gene promoter, such as Fos or Arc, drives CreERT2 expression selectively in recently active neurons. In resting neurons, the Fos/Arc promoter is inactive or only minimally active, CreERT2 is absent or expressed at very low levels, the STOP cassette remains intact, and TVA/RV-G are not expressed. In recently active neurons exposed to tamoxifen/4-OHT, CreERT2 is produced, translocates to the nucleus, and excises the floxed STOP cassette from the AAV-delivered responder/helper cassette. This permanently activates TVA and RV-G expression in neurons recruited during the defined activity window, allowing EnvA-RVΔG to infect these neurons as functionally defined starter cells.

Temporal control can also be added through inducible systems that restrict recombinase activity to a defined time window. The tetracycline-regulated systems operate as follows (Figure 3): in Tet-Off, a tetracycline-controlled transactivator protein (tTA)—a fusion of the bacterial Tet repressor with a transcriptional activation domain—drives transgene expression constitutively but is silenced by doxycycline, while in Tet-On a reverse variant (rtTA: reverse tetracycline-controlled transactivator) is inactive by default and switches on transcription only upon doxycycline binding, allowing precise pharmacological control over the timing and duration of TVA or RV-G expression (Gossen and Bujard, 1992). Such Tet-regulated control has also been incorporated into rabies-based helper AAV configurations to modulate the timing and level of helper-gene expression, providing an additional pharmacological layer for controlling tracing efficiency and toxicity (Jin et al., 2024). In tamoxifen-dependent Cre-ER (Cre recombinase fused to a modified estrogen receptor domain) systems, the starter cell expresses a fusion protein in which Cre recombinase is fused to a ligand-binding domain derived from a modified estrogen receptor that no longer responds to the endogenous hormone but retains sensitivity to the synthetic ligand tamoxifen (Figure 4A). In the absence of tamoxifen, the chimeric protein is retained in the cytoplasm through binding to heat-shock proteins; only after tamoxifen administration does it dissociate, translocate to the nucleus, and activate recombination, thereby conferring starter-cell competence only during the period of drug exposure (Feil et al., 1997).

### 5.3 Activity-dependent systems

**Figure 5:**
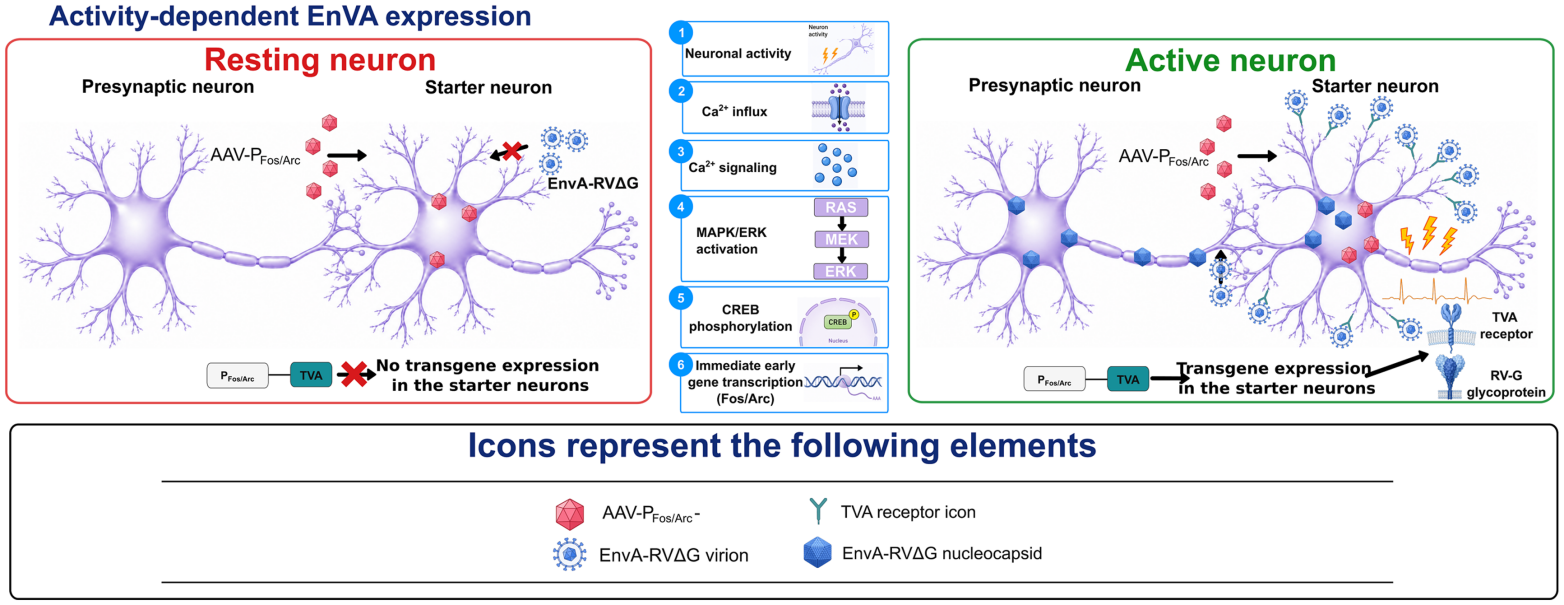
Activity-dependent starter cell targeting. In this system, TVA expression is placed under the control of an immediate early gene promoter (Fos or Arc), delivered to neurons by AAV (AAV-P_Fos/Arc_-TVA). In resting neurons (left), basal promoter activity is insufficient to produce functional TVA levels, and EnvA-RVΔG cannot infect. Neuronal activity (right) triggers a signaling cascade — Ca^2+^ influx → CaMK activation → MAPK/ERK activation → CREB phosphorylation → immediate early gene transcription (Fos/Arc) — that drives TVA expression on the cell surface, rendering recently active neurons selectively infectable by EnvA-RVΔG.

A further dimension is added by activity-dependent strategies such as TRAP (targeted recombination in active populations) in which Cre-ER is expressed under the control of an immediate early gene promoter—such as Fos or Arc, which are rapidly transcribed in active neurons (Figure 4B). Because tamoxifen opens only a brief time window for Cre-ER to enter the nucleus, Cre-mediated excision of a floxed (loxP-flanked) STOP cassette occurs only in neurons that are active during that period. This makes it possible to permanently switch on reporter or helper gene expression in neurons involved in a specific stimulus, behavior, or learning episode and to use them later as a functionally defined starter population for circuit tracing (Guenthner et al., 2013). In the CANE (Capturing Activated Neuronal Ensembles) approach, an AAV vector delivers a viral entry receptor—such as TVA—under the control of an activity-dependent promoter (Sakurai et al., 2016) (Figure 5). Neurons that are active during a defined behavioral experience transcribe the receptor and display it on their surface; a subsequently injected EnvA-pseudotyped virus can therefore infect only this recently active population, without requiring transgenic animals.

### 5.4 Projection-defined systems

**Figure 6:**
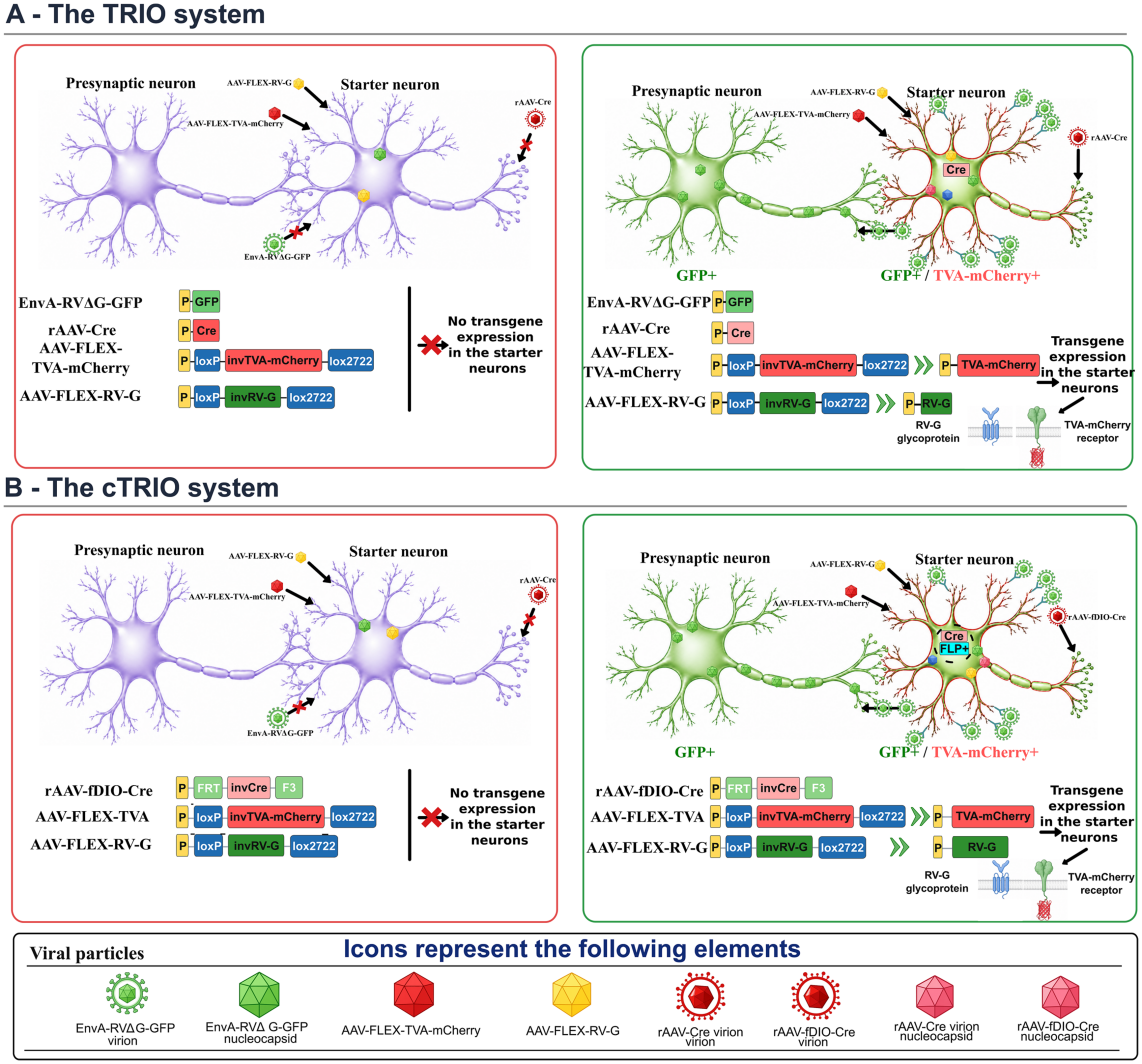
Projection-defined starter cell targeting for monosynaptic rabies virus tracing. Each panel compares the inactive state (left, red border) with the active tracing configuration (right, green border), showing both the neuronal outcome and the underlying expression cassette architecture. Viral particle icons are defined in the figure key. **(A)** The TRIO system. Three AAV vectors and one rabies virus vector are used: rAAV-Cre is injected into the target area and taken up by axon terminals, delivering Cre retrogradely to projection neurons in the source region. AAV-FLEX-TVA-mCherry (P–loxP–invTVA-mCherry–lox2722) and AAV-FLEX-RV-G (P–loxP–invRV-G–lox2722) are injected into the source region as Cre-dependent FLEX cassettes. EnvA-RVΔG-GFP, carrying GFP within the viral genome, is subsequently injected into the same region. In neurons that did not receive retrograde rAAV-Cre and therefore lack Cre (left), all FLEX cassettes remain in the inverted, inactive orientation; EnvA-RVΔG-GFP cannot infect cells lacking TVA. In projection neurons that took up rAAV-Cre from the target area and express Cre (right), recombination inverts both FLEX cassettes into the sense orientation, activating TVA-mCherry and RV-G expression. EnvA-RVΔG-GFP infects TVA-mCherry (mCherry fluorescent protein)-expressing starter cells and spreads retrogradely one synaptic step to presynaptic input neurons. Starter neurons are identified by co-expression of membrane-bound TVA-mCherry (red) and virally delivered GFP (green), appearing yellow in merged imaging channels; presynaptic input neurons, which lack TVA-mCherry but receive the virus retrogradely, are labeled green only. **(B)** The cTRIO system. The cassette architecture is identical to TRIO for AAV-FLEX-TVA-mCherry (P–loxP–invTVA-mCherry–lox2722) and AAV-FLEX-RV-G (P–loxP–invRV-G–lox2722). The sole difference is that the retrograde vector carries a Flp-dependent Cre construct, rAAV-fDIO-Cre (P–FRT–invCre–F3), instead of constitutively expressed Cre. In neurons lacking both recombinases (left), neither the fDIO-Cre cassette nor the FLEX helper cassettes are activated, and no gene expression occurs. Only neurons that both project to the target area—receiving rAAV-fDIO-Cre retrogradely—and express Flp from a transgenic Flp driver line (right) produce Cre, which in turn activates the FLEX-TVA-mCherry and FLEX-RV-G cassettes. This dual requirement restricts starter cell competence to neurons defined simultaneously by their projection target and their molecular identity. The fluorescent labeling outcome is identical to TRIO: starter neurons appear yellow (TVA-mCherry+ and GFP+) and presynaptic input neurons appear green only (GFP+).

TRIO (Tracing the Relationship between Input and Output) is used to identify the inputs to neurons defined by where they project (Figure 6A). First, a retrograde vector—rAAV, in which the “r” prefix denotes its retrograde-optimized capsid rather than a directional property of AAV in general, carrying a construct in which a strong ubiquitous promoter (such as EF1α or CAG) drives Cre recombinase expression (rAAV-Cre)—is injected into a target area. It is taken up by the axon terminals of neurons projecting to that area and transported retrogradely to their cell bodies; once inside the soma, the AAV genome is transcribed and Cre protein is produced, which then enters the nucleus and becomes active, thereby causing those projection neurons to express Cre without the vector crossing synapses. Next, two AAV helper vectors are injected into the brain region containing these projection neurons: one carrying a Cre-dependent FLEX-TVA-mCherry cassette (AAV-FLEX-TVA-mCherry: promoter – loxP – [inverted TVA-mCherry] – lox2722), in which mCherry is directly fused to TVA and, because TVA is a type I transmembrane protein, the fusion protein is membrane-anchored, fluorescently labeling the soma and dendrites of starter cells in red (Callaway and Luo, 2015) and one carrying a Cre-dependent FLEX-RV-G cassette (AAV-FLEX-RV-G: promoter – loxP – [inverted RV-G] – lox2722). In projection neurons that received rAAV and therefore express Cre, the recombinase inverts both transgenes into the sense orientation, activating TVA-mCherry and RV-G expression. EnvA-RVΔG-GFP is then injected into the same region, where it binds to TVA-mCherry on the soma and dendrites of starter cells and enters them. Because EnvA-RVΔG-GFP carries GFP (green fluorescent protein) in its genome, it expresses GFP in every neuron it enters — both in starter cells and, after retrograde spread, in their presynaptic input partners. Starter cells therefore carry both fluorescent signals: red from the membrane-bound TVA-mCherry and green from the virally expressed GFP, resulting in a yellow signal when the two channels are merged. Presynaptic input neurons, which receive EnvA-RVΔG-GFP retrogradely but were never transduced by the AAV helper vectors and therefore express neither TVA-mCherry nor RV-G, appear green only. Because those partners lack RV-G, further transsynaptic spread is blocked. The experiment thus labels three distinct neuronal populations: the injection site in the target area, from which the axon terminals of projection neurons took up rAAV-Cre; the starter neurons in the source region, identified by their yellow signal (red mCherry and green GFP); and their direct presynaptic inputs, labeled green only by EnvA-RVΔG-GFP.

In conditional TRIO (cTRIO), the AAV cassettes are identical to those used in TRIO—AAV-FLEX-TVA (Promoter – loxP – [inverted TVA] – lox2722) and AAV-FLEX-RV-G (Promoter – loxP – [inverted RV-G] – lox2722)—with the sole difference that the retrograde vector carries a Flp-dependent construct (rAAV-fDIO-Cre: Promoter – FRT – [inverted Cre] – F3) instead of a constitutively expressed Cre. This means that Cre is produced in a retrogradely labeled neuron only if that neuron itself expresses Flp—conferred by a transgenic Flp driver line in which Flp is expressed under a cell-type-specific endogenous promoter (Figure 6B). In this case, Cre is expressed only in neurons that both project to the target area and whose genome encodes Flp under a cell-type-specific promoter, typically marking a specific cell type. This dual requirement restricts the starter population to neurons defined simultaneously by their projection target and their molecular identity (Beier et al., 2015; Schwarz et al., 2015). Together, these approaches allow viral tracing to be targeted not only to molecularly defined cell types, but also to neurons defined by their recent activity, projection target, or combinations of these features. These strategies form a hierarchy of increasing restriction, from simple Cre-dependent targeting to intersectional, activity-defined, and projection-defined starter-cell selection.

## 6 Spatial and transcriptomic readouts of viral tracing

### 6.1 Molecular barcoding and transcriptomic integration

**Figure 7:**
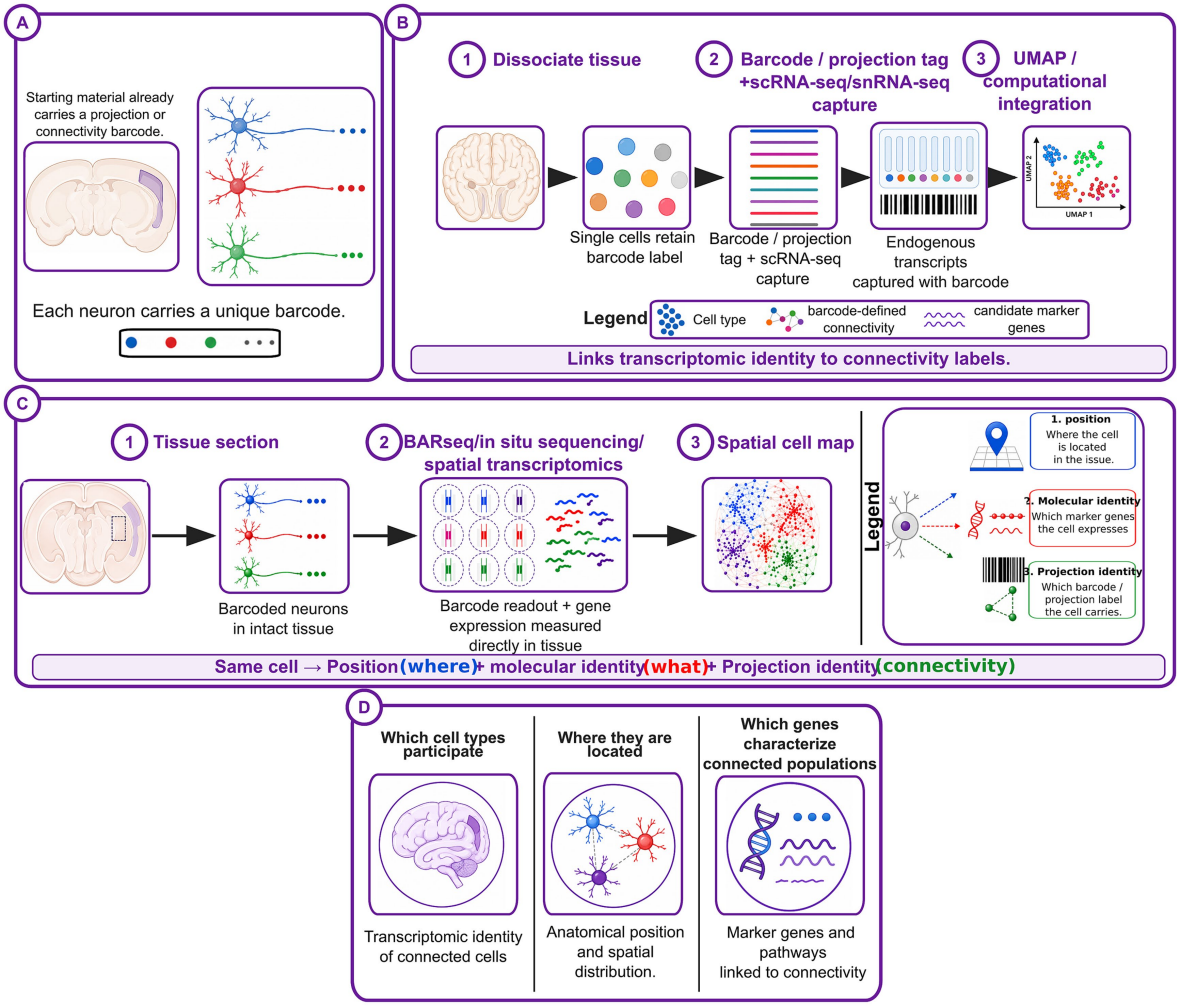
Molecular profiling of connectivity-defined neuronal circuits. **(A)** Connectivity-defined neurons are labeled by tracing or barcode-based strategies, so that each neuron or cell carries a projection/connectivity label. **(B)** In the dissociation-based single-cell route, labeled cells or nuclei are isolated and analyzed by scRNA-seq or snRNA-seq (single-nucleus RNA), allowing barcode or projection tags to be computationally linked to endogenous transcriptomes. **(C)** In the spatial *in situ* route, barcode readout and gene-expression measurements are performed directly in tissue, preserving anatomical position and local context. **(D)** These approaches identify the cell types, spatial distribution, marker genes, pathways, and potential circuit organization of connected neuronal populations.

Standard RV tracing labels all input neurons with the same fluorescent marker, making it impossible to determine which inputs connect to which individual starter neuron. Molecular barcoding addresses this by incorporating a unique short nucleotide sequence into each viral particle, so that every starter neuron and its presynaptic partners carry a distinct tag readable by sequencing, enabling connectivity to be mapped at single-cell resolution across many starter neurons simultaneously (Saunders et al., 2022; Zhang et al., 2024) (Figure 7A). A key challenge is ensuring that the barcode library is large and uniform enough for each starter neuron to receive a unique tag; recent optimizations have improved library diversity and uniformity while reducing background contamination, enabling reliable labeling of large numbers of starter neurons in a single experiment (Zhang et al., 2024). Transcriptomic approaches provide a complementary molecular readout of these virally traced circuits (Patiño et al., 2024). Combining barcoded RV with single-cell RNA sequencing (scRNA-seq)—in which dissociated cells are individually profiled for genome-wide gene expression—has made it possible to determine which transcriptomic cell types are connected and to analyze connectivity in relation to gene expression (Figure 7B). This approach, exemplified by the SBARRO (Synaptic Barcode Analysis by Retrograde Rabies ReadOut) method, showed that synaptic partner relationships can be inferred in a single experiment while simultaneously capturing genome-wide transcriptomes (Saunders et al., 2022). While powerful, scRNA-seq requires tissue dissociation and therefore removes native spatial context. *In situ* sequencing approaches address this by reading out both viral barcodes and endogenous transcripts directly within tissue sections, thereby preserving spatial context. BARseq (Barcoded Anatomy Resolved by Sequencing) is one such method, combining *in situ* barcode sequencing with detection of a selected panel of marker genes (Chen et al., 2019). BARseq was originally developed for projection mapping of non-virally barcoded neurons and later adapted to barcoded RV, allowing transsynaptic input relationships to be linked with molecular cell identity while preserving spatial information in tissue sections (Zhang et al., 2024) (Figure 7C). Complementary approaches such as MAPseq (Multiplexed Analysis of Projections by Sequencing) recover barcodes from dissected target regions at high throughput, but sacrifice spatial information (Kebschull et al., 2016). Together, these approaches identify the cell types, spatial distribution, marker genes, pathways, and potential circuit organization of connected neuronal populations (Figure 7D).

### 6.2 Whole-brain imaging

Modern viral tracing is increasingly combined with whole-brain imaging methods that make it possible to examine labeled circuits across the entire brain rather than in a limited number of tissue sections. Tissue-clearing methods such as CLARITY (Clear Lipid-exchanged Acryl-hybridized Rigid Imaging/Immunostaining/In situ hybridization-compatible Tissue hYdrogel) and iDISCO (immunolabeling-enabled three-dimensional imaging of solvent-cleared organs) make the brain transparent by removing lipids while preserving fluorescent signals and protein structure, allowing fluorescently labeled neurons and axons to be visualized deep within intact tissue (Chung et al., 2013; Renier et al., 2014). These cleared brains can then be imaged in three dimensions by light-sheet fluorescence microscopy, which illuminates only a thin plane of tissue at a time and thereby achieves high imaging speed with minimal photobleaching across large volumes. When the resulting images are aligned computationally to a reference brain atlas, labeled cells and projections can be assigned to defined anatomical regions and quantified across the whole brain in a standardized way (Pisano et al., 2021). The combination of viral tracing with tissue clearing and atlas-based registration has been particularly powerful for generating brain-wide input maps from defined starter populations, and for comparing connectivity patterns across experimental conditions or genotypes (Frankowski et al., 2022). An alternative that avoids the technical demands of clearing is serial section-based 3D reconstruction using automated slide scanning. When complete section series are digitized and registered to a reference atlas, labeled projections can be mapped with anatomical fidelity comparable to clearing-based approaches, while retaining full compatibility with conventional immunofluorescence protocols and avoiding depth-dependent antibody penetration artifacts (Oh et al., 2014).

## 7 From anatomy to function: integrative viral circuit analysis

### 7.1 Functional readouts: calcium and voltage imaging

**Figure 8:**
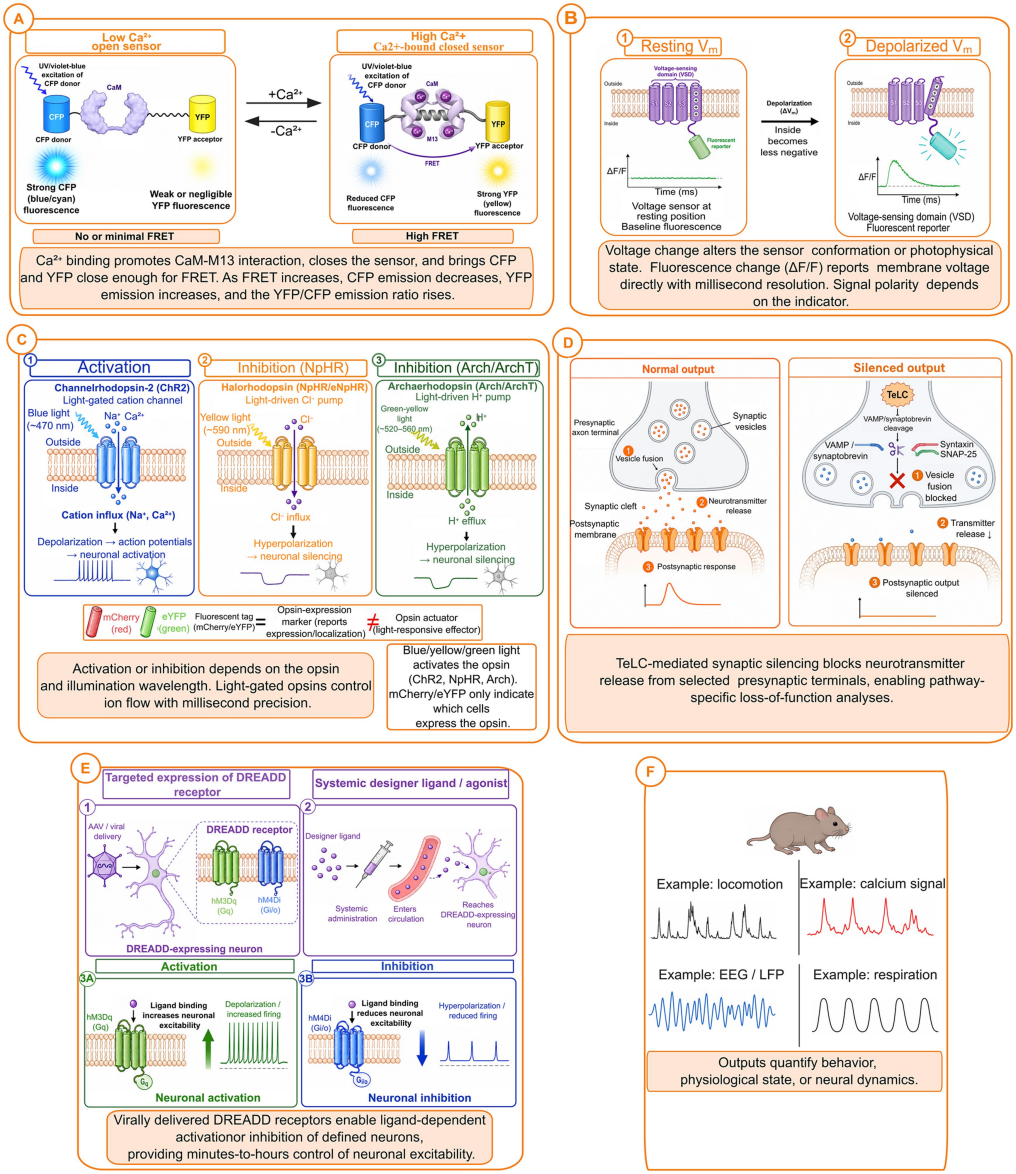
Functional interrogation of connectivity-defined neuronal circuits. **(A)** FRET (fluorescence resonance energy transfer)-based genetically encoded calcium indicators report intracellular Ca^2+^ through donor excitation and Ca^2+^-dependent energy transfer: low Ca^2+^ produces minimal FRET and dominant CFP (cyan fluorescent protein) emission, whereas Ca^2+^ binding promotes CaM–M13 interaction, sensor closure, increased CFP-to-YFP energy transfer, reduced CFP emission, increased YFP (yellow fluorescent protein) emission, and a higher YFP/CFP ratio. **(B)** Genetically encoded voltage indicators report membrane voltage directly through voltage-dependent conformational or photophysical changes that generate fluorescence changes with millisecond resolution; signal polarity depends on the specific indicator. **(C)** Optogenetic tools provide wavelength-specific control of neuronal activity: channel rhodopsin-2 (ChR2) mediates blue-light-driven cation influx and depolarization, whereas NpHR/eNpHR and Arch/ArchT mediate light-driven hyperpolarization through Cl^−^ influx or H^+^ efflux, respectively. Fluorescent tags such as mCherry or eYFP indicate opsin expression and localization but are not themselves the light-responsive actuator. **(D)** TeLC-mediated synaptic silencing blocks neurotransmitter release at selected presynaptic terminals by disrupting vesicle fusion, enabling pathway-specific loss-of-function analyses. **(E)** DREADD-based chemogenetics enables systemic ligand-dependent activation or inhibition of defined neurons over minutes to hours through virally delivered designer receptors such as hM3Dq or hM4Di. **(F)** Behavioral and physiological outputs, including locomotion, calcium signals, EEG/LFP activity, and respiration, provide systems-level readouts of circuit function. Together, these approaches transform viral circuit mapping from anatomical labeling into molecularly annotated, physiologically monitored, and causally testable circuit analysis.

Beyond identifying which neurons are connected, viral tracing can be combined with genetically encoded activity indicators to determine when and how those circuits are engaged. Early proof-of-principle studies showed that functional reporters can be incorporated directly into neurotropic tracer genomes, including PRV constructs expressing genetically encoded calcium indicators (GECIs) such as TN-L15 (Boldogkoi et al., 2009; Granstedt et al., 2009). As one example of calcium indicator design, FRET-based sensors report Ca^2+^-dependent conformational changes through altered donor–acceptor fluorescence, as schematized in Figure 8A (Miyawaki et al., 1997). It has been shown that retrograde PRV tracers carrying TN-L15 enabled two-photon optical monitoring of activity in retinal ganglion cells examined *ex vivo* after transsynaptic retrograde labeling from distant inoculation sites (Boldogkoi et al., 2009). Depending on the injection paradigm, neurons were labeled across one or multiple synaptic steps, demonstrating that transsynaptically identified populations can be functionally analyzed by optical methods after circuit-specific tracing. Because this strategy relied on optical readout rather than conventional single-cell electrophysiology, it also enabled the parallel monitoring of multiple identified neurons within a spatially intermingled population. The same study introduced genetically timed PRVs, in which an early membrane-associated green reporter was followed several hours later by a delayed red reporter, providing an internal readout of infection stage. In the combined sensor-timer configuration, calcium responses remained stable before red fluorescence appeared, whereas the onset of the red signal marked a more advanced stage of infection at which neuronal physiology could no longer be assumed to be fully intact. Thus, the early green signal defined the optimal recording window, while the later red signal served as a built-in warning that functional readouts should subsequently be interpreted with increasing caution. This general principle was later extended to RVΔG-based systems, which were adapted to express activity sensors, actuators, and recombinases, thereby enabling both monitoring and manipulation of connectivity-defined neuronal populations (Osakada et al., 2011). In parallel, AAV vectors have provided a complementary route for expressing activity indicators in genetically or projection-defined neuronal populations identified by retrograde, anterograde, or intersectional tracing strategies, including through retrograde AAV variants such as rAAV (Tervo et al., 2016).

GECIs—particularly the GCaMP family and the newer jGCaMP8 variants with ultra-fast kinetics—have become central tools for linking anatomical connectivity to activity dynamics (Chen et al., 2013; Zhang et al., 2023). AAV vectors are particularly useful for stable long-term expression during behavior, whereas RV-based approaches were initially constrained by toxicity. More recent tools have substantially reduced this limitation: RVΔG vectors expressing GCaMP have been used for *in vivo* functional imaging, and second-generation double-deletion RV systems have enabled longer-term calcium imaging of synaptically connected networks with markedly reduced cytotoxicity (Bouin et al., 2024; Jin et al., 2024). Red-shifted indicators such as jRCaMP and jRGECO further support two-color experiments by allowing one population to be imaged in green and another in red, while also improving compatibility with optogenetic stimulation paradigms by separating the excitation spectrum of the indicator from that of the optogenetic actuator (Dana et al., 2019).

Genetically encoded voltage indicators (GEVIs) extend this logic by providing a direct readout of membrane potential with substantially higher temporal resolution than calcium imaging (Figure 8B). While calcium imaging reports neuronal firing with a delay of tens to hundreds of milliseconds because calcium influx follows action potentials indirectly, voltage indicators track membrane voltage changes on a millisecond timescale, making them particularly well suited for resolving subthreshold synaptic potentials and precise spike timing. In practice, the GEVI is typically delivered by a separate AAV vector and targeted to the same neuronal population as the rabies tracer, so that connectivity-defined neurons express both the fluorescent circuit label and the voltage indicator simultaneously. Representative GEVIs include ASAP-family and ArcLight-type sensors, which have helped establish voltage imaging as a useful approach for examining how traced synaptic inputs are combined and processed in target neurons with millisecond resolution (Gong et al., 2015; Knöpfel and Song, 2019). Although technically demanding—because voltage signals are small and fast, requiring high-speed cameras and careful optical design—voltage imaging is increasingly feasible *in vivo* and offers access to circuit computations that calcium imaging cannot resolve.

### 7.2 Activation and silencing of traced circuits by optogenetics and chemogenetics

Viral tracing becomes especially powerful when combined with methods that test the causal roles of anatomically defined pathways. Optogenetics, in which light-sensitive proteins are expressed in genetically targeted neurons to allow their activation or inhibition with light, provides the most widely used framework for this purpose because it offers millisecond temporal precision (Boyden et al., 2005) (Figure 8C). In practice, this often involves Channelrhodopsin-based activation of traced neurons or their terminals to test whether anatomically identified pathways are sufficient to drive a given response or behavior. For example, optogenetic activation of distinct basolateral amygdala-to-bed nucleus of the stria terminalis projections was shown to bidirectionally regulate anxiety-like behavior, illustrating how pathway-specific manipulations can link circuit anatomy to behavioral output (Han et al., 2024). Beyond emotional behavior, optogenetic dissection of projection-defined circuits has been applied to movement control, reward learning, sensory processing, and many other systems, demonstrating the broad utility of combining anatomical tracing with causal intervention. Optogenetic actuators can be delivered by retrograde AAV vectors to projection-defined neurons, expressed in genetically defined starter populations through recombinase-dependent constructs, or restricted to neurons active during a particular behavioral epoch using activity-dependent promoters. Beyond activation-based manipulations, viral vectors can also deliver synaptic silencing tools such as tetanus toxin light chain (TeLC) to block neurotransmitter release from defined projections, enabling pathway-specific loss-of-function testing (Yamamoto et al., 2003) (Figure 8D).

Chemogenetic approaches provide a complementary strategy for manipulating traced cell populations over longer timescales (Figure 8E). Chemogenetics introduces genetically engineered receptors into neurons that respond exclusively to a synthetically designed small molecule—a designer ligand—that is otherwise inert in the brain and does not activate endogenous neurotransmitter receptors or respond to conventional drugs. This allows a defined neuronal population to be activated or inhibited pharmacologically, but with genetic precision. A widely used example is the DREADD (Designer Receptors Exclusively Activated by Designer Drugs) system, in which muscarinic acetylcholine receptors are mutated such that they no longer respond to their endogenous ligand acetylcholine, but instead respond selectively to a designer ligand, originally clozapine-N-oxide (CNO) and more recently deschloroclozapine (DCZ), which shows improved brain penetrance and lower off-target effects. The two principal DREADD variants—both engineered G-protein-coupled receptors derived from human muscarinic acetylcholine receptors—operate through distinct pathways: hM3Dq couples to G_q_, triggering phospholipase C activation, IP3/DAG signaling, and intracellular Ca^2+^ release, thereby depolarizing and activating the targeted neurons; hM4Di couples to G_i_, inhibiting adenylate cyclase and opening inwardly rectifying K^+^ channels, causing hyperpolarization and suppressing neuronal firing. Both are activated by systemic administration of the designer ligand, allowing remote pharmacological control of genetically defined circuit elements without the need for implanted optical fibers (Alexander et al., 2009). In the context of viral circuit tracing, DREADDs are typically delivered to connectivity-defined neuronal populations either by incorporating the DREADD transgene directly into the rabies virus genome, by including it in the AAV helper vector alongside TVA and RV-G, by injecting a separate Cre-dependent AAV-DREADD vector into the starter cell region after tracing is complete, or by using transgenic mouse lines in which a Cre-dependent DREADD is knocked into the genome and activated in the starter population by the same Cre driver used for tracing. This allows the same population identified by transsynaptic labeling to be subsequently activated or silenced in a behaving animal. Unlike optogenetics, chemogenetics does not require implanted optical fibers, making it more practical for targeting distributed or deep circuits defined by viral tracing, but at the cost of spatial and temporal precision. Chemogenetic activation or silencing of projection-defined or cell-type-specific neuronal populations has been applied across diverse systems—including dopaminergic circuits, prefrontal-subcortical pathways, and brainstem nuclei—to establish causal roles for connectivity-defined circuit elements in behavior (Alexander et al., 2009).

These multimodal combinations expand viral tracing from a labeling strategy into an analytical framework: barcoding and sequencing identify which molecularly defined cells are connected, tissue clearing and atlas registration reveal where these cells are positioned in the intact brain, and functional readouts (Figure 8F) or perturbations test how the mapped pathways contribute to circuit dynamics and behavior.

## 8 Challenges and limitations

### 8.1 Synaptic specificity and quantitative interpretation

The assumption that viral tracers spread specifically through synaptic connections is central to the interpretation of viral circuit mapping, yet this assumption should be treated with caution. For rabies virus, several observations are consistent with predominantly connection-dependent transfer: labeled neurons often follow known anatomical circuits, RVΔG spread is restricted to first-order presynaptic populations when RV-G is supplied only in starter cells, and labeling probability is related to synaptic contact number. However, these observations do not by themselves prove that all labeled neurons are reached exclusively through synaptic junctions. Alternative explanations, including transfer at sites of close membrane apposition, local leakage, or nonspecific uptake near the injection site or damaged processes, remain difficult to exclude experimentally (Beier, 2019; Rogers and Beier, 2021). Recent synapse-resolution analyses further indicate that rabies labeling is probabilistic: synapse number contributes to labeling probability, but many anatomically connected neurons remain unlabeled, and postsynaptic cell type or synaptic distance alone does not explain labeling efficiency (Patiño et al., 2022a).

Non-synaptic or nonspecific labeling can arise under several conditions. In polysynaptic alphaherpesvirus tracing, late survival times and high viral loads increase cytopathic damage, which can release infectious particles into the extracellular space and allow infection of nearby neurons that are not synaptically connected (Card et al., 1993; Fekete et al., 2018). In RVΔG-based systems, nonspecific uptake at sites of membrane damage, local leakage near the injection site, or uptake by axons of passage cannot be completely excluded, especially when viral titers are high or helper expression is leaky (Callaway and Luo, 2015; Rogers and Beier, 2021). For AAV-based transsynaptic tagging, synaptic vesicle release appears to contribute to transfer, but passive transcytosis or non-synaptic uptake may also occur, and distinguishing these mechanisms experimentally remains difficult (Zingg et al., 2017, 2020).

These limitations also constrain quantitative interpretation. Rabies-based input maps should not be treated as complete or unbiased inventories of all presynaptic partners. The absence of a labeled neuron does not prove the absence of a synaptic connection, and the number of labeled input cells cannot be interpreted directly as connection strength without appropriate normalization. Common metrics such as input fraction and convergence index depend strongly on starter-cell number and other experimental variables, making comparisons across experiments unreliable unless starter populations, viral titers, survival times, imaging pipelines, and statistical normalization are carefully matched (Tran-Van-Minh et al., 2023). Viral tracing therefore provides a weighted, tool-dependent sample of connectivity rather than an unbiased ground truth.

### 8.2 Multimodal integration constraints

Combining viral tracing with transcriptomic readouts introduces additional complications: RV infection itself alters host gene expression, with widespread downregulation of neuronal marker genes, which can interfere with transcriptomic cell type classification of labeled neurons—particularly when only small gene sets or single markers are used (Patiño et al., 2022b). Whole-brain imaging approaches based on tissue clearing are powerful but technically demanding; antibody penetration into large cleared samples can be incomplete, and fluorescence signal may degrade during the clearing process (Chung et al., 2013; Renier et al., 2014). Functional integration with GECIs and GEVIs is limited by the fact that calcium signals are substantially slower than the underlying electrical events, and GEVIs remain technically demanding with low signal-to-noise ratios *in vivo* (Chen et al., 2013; Gong et al., 2015). When combining functional readouts with optogenetics, spectral overlap between excitation and imaging wavelengths is a practical concern, as light used to drive actuators can bleed into indicator channels; this has motivated the development of red-shifted actuators and indicators (Dana et al., 2019). Chemogenetic approaches with DREADDs are less constrained by spectral overlap, but commonly used ligands can have off-target effects that confound behavioral interpretations (Alexander et al., 2009). Recombinase-based targeting is not without caveats: incomplete recombination in inducible Cre-ER systems can reduce labeling efficiency, and Cre expression itself can be toxic at high levels in certain cell types.

### 8.3 Toxicity and genomic stability

Toxicity remains a fundamental challenge across multiple platforms. Polysynaptic alpha-herpesviruses are highly cytopathic by design; their replication ultimately kills infected neurons and can trigger strong innate immune responses (Fekete et al., 2018), limiting experiments to relatively short time windows and potentially confounding physiological measurements. First-generation RV, while more controlled in its spread, induces progressive dysfunction and death of labeled neurons within approximately two weeks (Wickersham et al., 2007a; Reardon et al., 2016). Self-inactivating and double-deletion mutants have made substantial progress toward non-toxic tracing (Chatterjee et al., 2018; Ciabatti et al., 2023; Jin et al., 2024), but they introduce additional layers of genetic control that must be carefully tuned, and questions remain about long-term genomic stability (Ciabatti et al., 2023; Jin et al., 2023). Additionally, SiR preparations require careful quality control to minimize the fraction of virions that have already lost the PEST attenuation element before injection.

### 8.4 Cell-type tropism and species constraints

A further limitation is that viral tracers introduce nontrivial biases in cell-type tropism. PRV Bartha, HSV-1 H129, different RV strains, and different AAV serotypes each show characteristic preferences for particular neuronal subclasses and species, and these preferences may vary with age, injection route, and the presence of specific receptors or helper constructs (Pomeranz et al., 2005; Callaway and Luo, 2015; Xu et al., 2020). Matching the tool to the biological question—retrograde versus anterograde, mono- versus polysynaptic, fast versus long-term, high versus low toxicity—is therefore essential, and no single virus is universally optimal (Xu et al., 2020).

### 8.5 Experimental controls and validation

Because the monosynaptic RV system relies on multiple interdependent components—RV-G deletion, AAV-delivered TVA and RV-G, and EnvA pseudotyping—each step must be validated independently to rule out artifactual labeling. Recommended controls include: (1) omitting TVA to confirm that EnvA-RVΔG cannot infect cells without its receptor; (2) omitting RV-G to verify that the virus cannot spread transsynaptically beyond starter cells; (3) testing for leaky TVA or RV-G expression from Cre-dependent AAVs in the absence of Cre; and (4) assessing background labeling from non-specific viral uptake. Multi-animal replication is essential given the sensitivity of the system to small variations in viral titer, injection placement, and recombinase expression levels.

### 8.6 Practical guidance for tool selection

Choosing the most appropriate viral tracing strategy requires matching the tool to the biological question, because no single tracer is suitable for all circuit-mapping experiments. If the goal is to identify direct presynaptic inputs to a genetically or projection-defined starter population, EnvA-pseudotyped RVΔG systems remain the most widely used option, but their output should be interpreted as a probabilistic sample of direct inputs rather than a complete quantitative connectome. If long-term functional access is required, lower-toxicity SiR, RVΔGL, or ΔL platforms are preferable, although they require additional quality control and may involve lower expression levels or more complex helper configurations.

For output mapping, H129-derived systems, H129 amplicons, AAV-based transsynaptic tagging, and synthetic platforms such as ATLAS provide complementary but non-equivalent solutions. H129-based tools offer strong labeling and access to output pathways, but directionality and synaptic order depend critically on survival time and viral design. AAV1/AAV9-based approaches are experimentally simple and compatible with Cre-dependent genetic systems, but they are less efficient and less strictly synapse-specific. ATLAS and related synthetic systems reduce toxicity and introduce activity dependence, but their efficiency, generality across cell types, and scalability still require further validation.

Polysynaptic PRV and HSV-1 tracers remain most useful when the biological question concerns multi-step circuit architecture rather than direct monosynaptic connectivity. Their main advantages are the ability to reveal hierarchical pathways across several synaptic stations and the large transgene capacity of alphaherpesvirus genomes, which facilitates the incorporation of complex reporter and effector constructs. However, these strengths come at the cost of increasing ambiguity over synaptic order, directionality, and nonspecific spread at later time points. Across all platforms, the most reliable circuit interpretations come from convergent evidence: viral tracing should ideally be combined with independent anatomical, electrophysiological, transcriptomic, imaging, or functional perturbation approaches.

## 9 Outlook

Viral circuit mapping has evolved from early polysynaptic labeling methods into a diverse and increasingly precise toolkit of mono- and polysynaptic approaches tailored to specific cell types, directions of spread, and experimental goals. Classical PRV and HSV-1 tracers remain valuable for revealing larger circuit organization, while engineered RV systems now provide more precise access to direct presynaptic inputs. At the same time, newer anterograde approaches—including H129-derived tracers, AAV-mediated transsynaptic tagging, and synthetic platforms—are expanding the mapping of output pathways with greater precision and interpretability. The field is moving toward integrated circuit analysis approaches that combine anatomical tracing, molecular profiling, functional recording, and causal manipulation within the same experimental framework, linking connectivity to cell identity, physiological dynamics, and behavioral relevance. Barcoded RV vectors (Zhang et al., 2024), tissue clearing (Chung et al., 2013), light-sheet imaging, calcium and voltage imaging (Chen et al., 2013; Gong et al., 2015), opto- and chemogenetics (Boyden et al., 2005; Alexander et al., 2009), activity-dependent tagging, and single-cell transcriptomics are making such integrative designs increasingly feasible. Important technical challenges remain: viral payload capacity limits the simultaneous delivery of multiple components; spectral overlap between imaging and stimulation wavelengths complicates combined experiments, driving the development of red-shifted optogenetic actuators and genetically encoded indicators that operate at longer wavelengths compatible with calcium or voltage imaging (Dana et al., 2019); and co-expression of multiple tools may itself perturb circuit physiology. Successful integration will therefore require careful experimental design together with continued improvements in indicators, opsins, and viral platforms.

Looking further ahead, the integration of viral tracing with *in vivo* CRISPR (clustered regularly interspaced short palindromic repeats) screening—using AAV-delivered guide RNA libraries to perturb gene function in connectivity-defined neuronal populations—holds promise for systematically identifying genes required for circuit formation, maintenance, or function (Santinha et al., 2023). Future progress will depend on safer and more genetically precise monosynaptic systems, tighter control of anterograde and polysynaptic spread, and labeling strategies that are easier to interpret functionally.

Within this broader landscape, alphaherpesvirus-based tracers may retain particular value. Attenuated PRV strains remain attractive for polysynaptic applications because they are relatively easy to use experimentally and can be engineered for conditional or monosynaptic tracing (Xu et al., 2020). Simultaneous infection with spectrally distinct recombinant strains, as exemplified by the Rainbow virus approach (Boldogkoi et al., 2009), enables parallel mapping of independent circuit elements and identification of neurons receiving convergent inputs. If transneuronal spread can be constrained with sufficient precision, the incorporation of fluorescent activity markers and optogenetic effectors could extend polysynaptic tracing to the functional analysis of multisynaptic networks. Herpesviruses are particularly well suited to this purpose because their large transgene cargo capacity can accommodate complex multi-component genetic payloads, offering a route toward functional access across larger circuits that monosynaptic tools cannot provide.

A complementary strategy is to regulate viral spread across multiple synapses through viral factors supplied by the host cell. Proof-of-principle studies in cell culture have shown that PRV mutants unable to replicate on their own can be restored *in trans* by supplying the missing essential viral functions, such as IE180 (Wu et al., 2014). Although its *in vivo* implementation remains limited, this strategy provides a conceptual framework for more precisely controlled polysynaptic tracing. A related translational frontier is the extension of AAV-based neural tools toward human disease. AAV-mediated gene delivery has already entered clinical use in selected neurological disorders, and tracing-inspired viral strategies are beginning to inform neuro-oncology, where retrograde tools have been adapted to study neuron–glioblastoma interactions in patient-derived models. These developments raise important questions regarding off-target transduction, immune responses, and unintended perturbation of neural function that will need to be addressed as such technologies move closer to translational use. Beyond technical concerns, the use of viral tools for neural access in human contexts also raises ethical questions regarding long-term biosafety, the durability and limited reversibility of neuronal gene delivery, and the dual-use implications of technologies designed to access or modify defined neural circuits.

More broadly, scaling viral circuit mapping toward connectomics-level throughput and extending it beyond mice will require automated sampling, large-scale imaging, barcode-based strategies, interoperable data resources, and careful validation of viral tropism and translational constraints across species. Extension to non-human primates will require careful validation, because viral tropism, transsynaptic efficiency, and immune responses may differ substantially from those observed in rodents (Nassi and Callaway, 2006; Lyon et al., 2010; Briggs et al., 2016). The continued integration of anatomical tracing with molecular profiling and functional interrogation will be essential for moving viral circuit analysis beyond connectivity maps toward a more mechanistic understanding of circuit operation.

## Glossary

AAV: adeno-associated virus
AAV-FLEX-TVA: inverted TVA expressing AAV
AAV-FLEX-RV-G: inverted RV-G expressing AAV
AAV-RV-G: RV-G-expressing AAV
AAV-TK: thymidine kinase-expressing AAV
AAV-TVA: TVA gene-expressing AAV
AAV-TVA/RV-G: TVA- and RV-G-expressing AAV
ATLAS: anterograde transsynaptic label based on antibody-like sensors
BAC: bacterial artificial chromosome
BARseq: barcoded anatomy resolved by sequencing
CANE: capturing activated neuronal ensembles
CFP: cyan fluorescent protein
ChR2: channel rhodopsin-2
CLARITY: clear lipid-exchanged acryl-hybridized rigid imaging/immunostaining/*in situ* hybridization-compatible tissue hydrogel
Cre: Cre recombinase
Cre-ER / Cre-ERT2: Cre recombinase fused to a modified estrogen receptor domain
CRISPR: clustered regularly interspaced short palindromic repeats
cTRIO: conditional TRIO
DIO: double-floxed inverted orientation
DREADD: designer receptors exclusively activated by designer drugs
EnvA: avian sarcoma and leukosis virus envelope protein A
EnvA-RVΔG: EnvA-pseudotyped RVΔG
fDIO: Flp-dependent double-floxed inverted orientation
FLEX: flip-excision cassette
Flp: flippase recombinase
FRET: fluorescence resonance energy transfer
FRT: Flp/flippase recognition target
GECI: genetically encoded calcium indicator
GEVI: genetically encoded voltage indicator
GFP: green fluorescent protein
H129Amp: H129 amplicon
HSV-1: herpes simplex virus type 1
iDISCO: immunolabeling-enabled three-dimensional imaging of solvent-cleared organs
loxP: locus of crossing-over in bacteriophage P1
MAPseq: multiplexed analysis of projections by sequencing
mCherry: mCherry fluorescent protein
PEST: Pro-Glu-Ser-Thr-rich sequence
PRV: pseudorabies virus
rAAV: a retrograde-optimized AAV variant
rAAV-Cre: Cre-expressing rAAV
rtTA: reverse tetracycline-controlled transactivator
RV: rabies virus
RV-G: rabies virus glycoprotein
RV-G helper AAV: RV-G gene-expressing AAV
RVΔG: glycoprotein-deleted rabies virus
RVΔGL: double-deletion mutant rabies virus (glycoprotein and polymerase gene deletions)
SBARRO: synaptic barcode analysis by retrograde rabies readout
scRNA-seq: single-cell RNA sequencing
snRNA-seq: single-nucleus RNA sequencing
SiR: self-inactivating rabies virus
TELC: tetanus toxin light chain
TK: thymidine kinase
TRAP: targeted recombination in active populations
TRE: tetracycline response element
TRIO: tracing the relationship between input and output
tTA: tetracycline-controlled transactivator
TVA: tumor virus receptor A
TVA/RV-G helper AAV: TVA- and RV-G-expressing AAV
YFP: yellow fluorescent protein
